# Antimicrobial Drug Use and Methicillin-resistant *Staphylococcus aureus*, Aberdeen, 1996–2000

**DOI:** 10.3201/eid1008.020694

**Published:** 2004-08

**Authors:** Dominique L. Monnet, Fiona M. MacKenzie, José María López-Lozano, Arielle Beyaert, Máximo Camacho, Rachel Wilson, David Stuart, Ian M. Gould

**Affiliations:** * Statens Serum Institut, Copenhagen, Denmark;; † Aberdeen Royal Infirmary, Aberdeen, Scotland;; ‡ Hospital Vega Baja, Orihuela (Alicante), Spain;; § University of Murcia, Murcia, Spain

**Keywords:** MRSA, outbreak, antimicrobial utilization, antibiotic use, macrolide, third-generation cephalosporin, fluoroquinolone, time-series analysis, dynamic modeling, research

## Abstract

Similar to many hospitals worldwide, Aberdeen Royal Infirmary has had an outbreak of methicillin-resistant *Staphylococcus aureus* (MRSA). In this setting, the outbreak is attributable to two major clones. The relationships between antimicrobial use and MRSA prevalence were analyzed by time-series analysis. From June 1997 to December 2000, dynamic, temporal relationships were found between monthly %MRSA and previous %MRSA, macrolide use, third-generation cephalosporin use, and fluoroquinolone use. This study suggests that use of antimicrobial drugs to which the MRSA outbreak strains are resistant may be an important factor in perpetuating the outbreak. Moreover, this study confirmed the ecologic effect of antimicrobial drug use (i.e., current antimicrobial use) may have an effect on resistance in future patients. Although these results may not be generalized to other hospitals, they suggest new directions for control of MRSA, which has thus far proved difficult and expensive.

## Abstract 2

Relationships between antimicrobial use and MRSA prevalence are analyzed in Aberdeen, Scotland.

Antimicrobial drug resistance occurs in hospitals worldwide. One of the most globally important microorganisms is methicillin-resistant *Staphylococcus aureus* (MRSA), which now causes more than 40% of all *S. aureus* bacteremias in the United Kingdom (1). Measures to control MRSA outbreaks have concentrated on transmission of the organism and prospective screening for carriage, in combination with general infection control measures such as patient isolation, use of barrier precautions, and environmental decontamination (2). Eradicating MRSA colonization has also been used to curb the spread of MRSA. Despite these measures, incidences of MRSA continue to rise (2,3). Guidelines for controlling MRSA in hospitals rarely include information on controlling antimicrobial use, possibly because relatively little data quantify the relationships between antimicrobial use and MRSA rates, especially in outbreak situations (4–8). To date, mathematical modeling has predicted that the effect of antimicrobial prescribing patterns in an outbreak situation is likely to be slight (9).

Epidemic MRSA type 15 (EMRSA-15) is presently the most common clone in the United Kingdom, followed by EMRSA-16, both of which are termed "super-clones" because of their potential for spreading nationally and internationally (10). Compared to other MRSA in the United Kingdom, EMRSA-15 and EMRSA-16 are more successful at surviving, colonizing, and spreading in the hospital environment (11). Both clones are typically resistant to all β-lactams, macrolides, and fluoroquinolones (10). The northeast of Scotland has seen a rapid spread of EMRSA-16 and, to a lesser extent, of EMRSA-15 during the last 7 years after they first emerged in the area's main teaching hospital, Aberdeen Royal Infirmary.

We investigate the dynamics of the MRSA outbreak at Aberdeen Royal Infirmary and possible relationships between MRSA prevalence and antimicrobial drug use, by time-series analysis. Time-series analysis creates a mathematical model to fit a series of dynamic observations to forecast future behavior on the basis of retrospective behavior. Unlike other statistical methods that assume observed data to be independent, time-series analysis takes into account the stochastic dependence of consecutive observations or autocorrelation (12,13). This method is appropriate when data are measured repeatedly at equal intervals for long periods and when these intervals are much shorter than the study period. Time-series analysis has been applied in medical specialties such as endocrinology, cardiology, environmental medicine, and the study of chronic diseases (13). The analysis of interrupted time-series or intervention analysis is considered the strongest quasi-experimental method to ascertain the longitudinal effect of healthcare interventions (13–15). Additionally, extensions of this method, e.g., transfer function modeling and econometric dynamic modeling, can take into account external factors that may influence the target series over time and can demonstrate a temporal relationship between these external factors and the target series (13–15). Because series of antimicrobial drug use data and resistance data always show an autocorrelation, this method has been proposed by López-Lozano et al. to study the relationship between antimicrobial drug use and resistance (16).

## Materials and Methods

Aberdeen Royal Infirmary is a 1,200-bed tertiary referral hospital covering a population of approximately 500,000. It comprises various medical and surgical specialties and is close to other specialized hospitals. For each month of the study period, January 1, 1996, to December 31, 2000, numbers of inpatient-days per ward were obtained from the hospital's admission department. During the study period, all *S. aureus* isolated were screened for susceptibility to methicillin by the comparative disc susceptibility test method on nutrient agar at 30°C with 48 h incubation (17). Methicillin resistance was confirmed by carrying out an Etest MIC. Susceptibility of the *S. aureus* isolates to a range of additional antimicrobial drugs was established by the comparative disc test method (17). Additionally, the Aberdeen MRSA outbreak was investigated by the Scottish MRSA Reference Laboratory, which conducted independent confirmation and genotyping. The Reference Laboratory carried out multiplex polymerase chain reaction (PCR) with primers to *mec*A, *nuc*, rRNA, 16S rRNA (18–20), and pulsed-field gel electrophoresis (PFGE) typing of *Sma*I digested DNA (21).

Monthly data for all *S. aureus* on which antimicrobial drug susceptibility tests were carried out were exported from the clinical microbiology information system into a database. Information stored included patient identifier, hospital, ward, specimen type, and antimicrobial drug–susceptibility pattern. Because we did not systematically and uniformly search for MRSA carriers, isolates obtained from surveillance screening were excluded. Only the first *S. aureus* isolate from each patient within 7 days was exported from the clinical microbiology laboratory information system into an Access (Microsoft, Redmond, WA) database. Variations in the antimicrobial susceptibility pattern of *S. aureus* isolates from the same patient within the 7-day period were not considered. From these data, the monthly prevalence of MRSA isolates was calculated as a percentage, where the denominator was the total number of *S. aureus* tested for methicillin resistance.

Monthly quantities of all antimicrobial drugs delivered to each hospital ward during the study period were exported from the pharmacy information system and stored both at the individual antimicrobial drug and class level in an Access (Microsoft) database. Antimicrobial drug use was expressed as a number of defined daily doses (DDDs) per 1,000 patient days, where the DDD for each antimicrobial drug was defined by the World Health Organization (WHO) (22). As in most hospitals, data on patient exposure to antimicrobial drugs were not available at Aberdeen Royal Infirmary. For a specific antimicrobial drug class, however, the number of DDDs approximates the average number of patients exposed to an antimicrobial drug from this class each day. This measurement is the unit WHO recommends to express ecologic pressure attributable to antimicrobial drugs (23).

Time-series analysis was carried out to explore the relationships between each antimicrobial drug use series and the %MRSA series. For this purpose, linear transfer function models were built according to the identification method proposed by Pankratz (15). This analysis was completed by a graphic exploration of the series. Line plots at monthly time intervals were produced for the %MRSA and for use of each antimicrobial drug class to visualize their evolution over time and to confirm the relationships between %MRSA and antimicrobial drug use.

Once the basic characteristics (i.e., autocorrelation, seasonality, and general trend) of each of the %MRSA and antimicrobial drug use series were established, a multivariate analysis was performed to quantify the relationships between use of several antimicrobial classes and %MRSA through the use of econometric dynamic time-series modeling techniques (14,24,25). Specifically, polynomial distributed lag (PDL) modeling was used to detect and quantify lagged effects of antimicrobial drug use on %MRSA. The details of the modeling technique are presented in the Appendix. For the purposes of this study, data were analyzed with Eviews 4.0 (Quantitative Micro Software, Irvine, California, USA).

## Results

**Figure 1:**
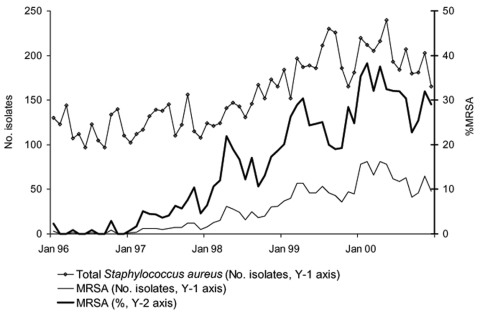
Evolution of the monthly number of clinical nonduplicate *Staphylococcus aureus* and methicillin-resistant *S. aureus* (MRSA) isolates and monthly %MRSA, Aberdeen Royal Infirmary, January 1996–December 2000.

From January 1996 through December 2000, the clinical microbiology laboratory isolated 9,441 nonduplicate, nonsurveillance *S. aureus*, including MRSA and methicillin-susceptible *S. aureus* (MSSA), from 6,412 hospitalized patients. Numbers ranged from 97 to 241 *S. aureus* isolates per month and demonstrated no seasonal patterns (Figure 1). The annual %MRSA from 1996 to 2000 were 0.6, 5.0, 14.9, 24.1, and 31.9, respectively. MRSA were rarely isolated before December 1996; after that date, a sustained increase was observed, with marked peaks of %MRSA observed in April 1998 (22%), April 1999 (30.5%), and February 2000 (38.2%) (Figure 1). Basic time-series analysis techniques and graphic exploration showed a spring seasonal variation of MRSA but no such seasonal variation for MSSA (Figure 1). From 1997 to 2000, the epidemic clones, EMRSA-16 and EMRSA-15, represented 80.0% and 15.4%, respectively, of 584 MRSA strains submitted for genotyping to the Scottish MRSA Reference Laboratory. Both clones were typically resistant to all β-lactams, macrolides, and fluoroquinolones but otherwise susceptible to other agents tested. The percentage of co-resistance to other antimicrobial drugs in all nonduplicate, nonsurveillance MRSAs (EMRSA-16, EMRSA-15, and other MRSA) isolated at Aberdeen Royal Infirmary during the outbreak is presented in Table 1. From 1996 to 2000, the annual use of systemic antibacterial agents showed little variation: 837, 953, 919, 963, and 938 DDD/1,000 patient-days, respectively. However, major variations occurred in the monthly use and seasonality of individual classes of antimicrobial drugs (Table 2).

**Table 1:** Antimicrobial drug coresistance in methicillin-resistant *Staphylococcus aureus* (MRSA) isolates and in methicillin-susceptible *S. aureus* (MSSA), Aberdeen Royal Infirmary, 1997–2000

Antimicrobial drug	MRSA isolates		MSSA isolates			
	No. tested for coresistance	No. resistant (%)	No. tested for coresistance	No. resistant (%)	Risk ratio	p value
Ciprofloxacin	1,218	1,195 (98.1)	515	183 (35.5)	13.4	< 0.0001
Clindamycin	2,722	2,666 (97.9)	7,715	956 (12.4)	89.6	< 0.0001
Erythromycin	2,721	2,669 (98.1)	7,701	1,115 (14.5)	90.0	< 0.0001
Fusidic acid	2,736	36 (1.3)	7,798	636 (8.2)	0.20	< 0.0001
Gentamicin	1,350	11 (0.8)	3,276	44 (1.3)	0.68	NS^a^
Mupirocin	2,514	154 (6.1)	5,180	99 (1.9)	1.92	< 0.0001
Rifampin	1,005	62 (6.2)	72	8 (11.1)	0.95	NS
Tetracycline	997	109 (10.9)	468	94 (20.1)	0.76	< 0.0001
Trimethoprim	1,060	18 (1.7)	0	–	–	–

^a^NS, nonsignificant.

**Table 2:** Characteristics of the monthly antimicrobial use time series, January 1996–December 2000

Antimicrobial drug class	Average monthly use^a^ (minimum–maximum)	Trend^b^	Seasonality^c^
Combinations of penicillins with β-lactamase inhibitors	228.6 (119.9–334.9)	Upward	Yes (0.294)
β-lactamase resistant penicillins	116.1 (49.1–202.1)	No	No
Macrolides	90.2 (32.7–177.9)	Upward	Yes (0.371)
Penicillins with extended spectrum	90.1 (43.9–177.4)	No	No
Third-generation cephalosporins	62.5 (43.8–103.1)	Upward	Yes (0.226)
β-lactamase-sensitive penicillins	54.6 (0–110.5)	No	No

Combinations of sulfonamides and trimethoprim, including derivatives	52.9 (0–86.8)	No	No
Fluoroquinolones	51.9 (19.4–87.5)	Upward	No
Second-generation cephalosporins	32.9 (5.3–87.1)	Downward	No
Other antibacterial drugs^d^	32.7 (16.3–45.9)	Upward	No
Tetracyclines	30.9 (0–63.4)	Downward	No
Aminoglycosides	24.8 (11.8–44.1)	Upward	Yes (0.236)
Glycopeptides	13.5 (4.6–25.5)	Upward	No
Lincosamides	6.1 (0–15.7)	Upward	Yes (0.208)
First-generation cephalosporins	5.2 (0.7–14.5)	No	No
Carbapenems	4.0 (0–8.5)	No	No

^a^Defined daily doses (DDD) per 1,000 mean patient-days. ^b^Based on regression of the series on time (according to the results of Dickey-Fuller unit root tests, none of the series needed to be differenced). ^c^Autocorrelation of order 12, based on the correlogram and the partial correlogram. When seasonality was present, the figure in parenthesis indicates the estimated autocorrelation of order 12, i.e., the correlation between antimicrobial use on a given month and use on the same month 1 year before. ^d^Amphenicols, monobactams, other quinolones, imidazoles, fusidic acid, and nitrofurantoin derivatives.

**Figure 2:**
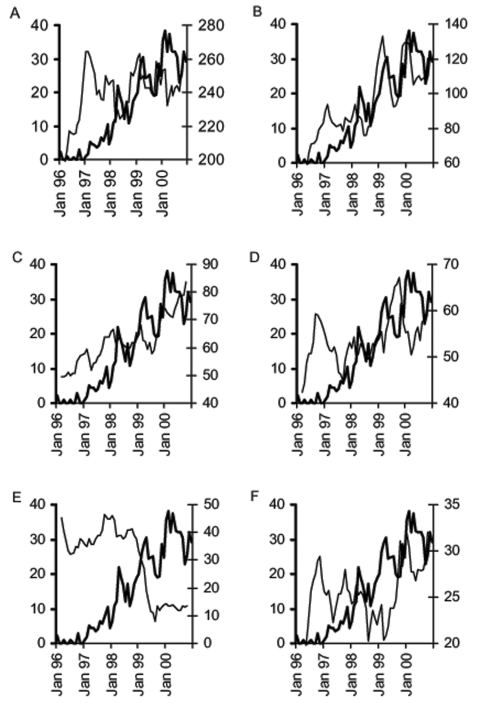
Examples of graphic exploration of the relationship between the monthly % methicillin-resistant *Staphylococcus aureus* (%MRSA) and the monthly use of individual classes of antimicrobials, Aberdeen Royal Infirmary, January 1996–December 2000 (THICK LINE, %MRSA; THIN LINE, Antimicrobial use, 5-month moving average, right Y-axis); A) penicillins with β-lactamase inhibitors, B) macrolides, C) third-generation cephalosporins, D) fluoroquinolones, E) tetracyclines, and F) aminoglycosides.

**Figure A1:**
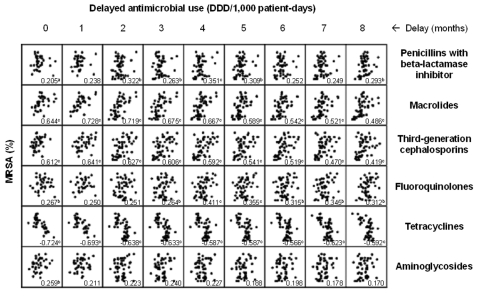
Examples of correlations between the monthly % methicillin-resistant *Staphylococcus aureus* (MRSA) and antimicrobial use without delay and with a 1- to 8-month delay, Aberdeen Royal Infirmary, January 1996–December 2000. ^a^Two-tailed Pearson correlation coefficient. ^b^Correlation significant at the 0.05 level. ^c^Correlation significant at the 0.01 level.

Time-series analysis showed that %MRSA had a relationship with the use of many antimicrobial drug classes. The relationship was strongest for macrolides, fluoroquinolones, and penicillins with β-lactamase inhibitors, whereas other classes showed a significant but weaker relationship (Table 3). Graphic exploration confirmed these findings and pointed at third-generation cephalosporin use as another series to be introduced in the initial multivariate model (Figure 2). We also examined scatter plots and correlations of %MRSA with use of individual classes of antimicrobial drugs with up to 8-month delays (Figure A1). However, this last approach proved less useful than time-series analysis, and graphic exploration of the time series in identifying relationships and optimal delays between antimicrobial drug use and %MRSA and could be misleading. For example, scatter-plots and correlations showed an inverse correlation between MRSA and tetracycline use. However, graphic exploration showed that this correlation reflected opposite general trends rather than monthly parallel variations between these two variables (Figure 2).

**Table 3:** Summary of transfer function models explaining the monthly %MRSA by use of each antimicrobial drug class^a^

Antimicrobial class^b^	Average delay (months)	Direction of effect^c^	p value	R^2 d^
Combinations of penicillins with β-lactamase inhibitors	2 4	Positive Positive	0.04 0.01	0.92
β-lactamase–resistant penicillins	0 6	Negative Positive	0.02 0.002	0.90
Macrolides	1	Positive	0.0001	0.93
Penicillins with extended spectrum	1	Positive	0.03	0.91
Third-generation cephalosporins	1	Positive	0.04	0.90
β-lactamase–sensitive penicillins	6	Positive	0.04	0.89
Combinations of sulfonamides and trimethoprim, including derivatives	4	Positive	0.02	0.90
Fluoroquinolones	4	Positive	0.0004	0.92
Second-generation cephalosporins		No relationship		
Other antibacterials^e^	0	Positive	0.002	0.91
Tetracyclines	4 7	Positive Negative	0.03 0.0007	0.91
Aminoglycosides		No relationship		
Lincosamides	7	Positive	0.02	0.89
First-generation cephalosporins		No relationship		
Carbapenems	3	Positive	0.03	0.90

^a^MRSA, methicillin-resistant *Staphylococcus aureus*. ^b^Glycopeptide use is not presented in this table because it showed an inverse relationship with %MRSA. In other words, %MRSA explained the monthly variations of glycopeptide use and not the reverse (Discussion). ^c^Positive direction of effect: increase in antimicrobial use results in increase in %MRSA and inversely. Negative direction of effect: increase in antimicrobial use results in decrease in %MRSA and inversely. ^d^All models include the variable %MRSA with a 1-month delay and a p value < 0.0001. ^e^Amphenicols, monobactams, other quinolones, imidazoles, fusidic acid, and nitrofurantoin derivatives.

A multivariate PDL model was built to relate %MRSA with use of these classes of antimicrobial drugs. The final model included previous monthly %MRSA as well as use of macrolides, third-generation cephalosporins, and fluoroquinolones as independent variables responsible for variations in %MRSA (Table 4). The greatest total effect of antimicrobial drug use on the %MRSA was found within the first two or three significant lag periods, after which the effect progressively decreased to reach nonsignificant values a few months after the end of the direct effect.

**Table 4:** Estimated multivariate polynomial distributed lag (PDL) model for monthly %MRSA (R^2^=0.902)^a^

Explaining variable	Lag (mo.)	Direct effect^b^			Indirect effect^c^	Sum of both effects^d^		
		Coeff	T-stat	p	Coeff	Coeff^e^	T-stat	p
%MRSA	1	0.420	3.96	0.0003				
Macrolide use								
Each month	1	0.083				0.083	4.02	0.0003
	2	0.055			0.035	0.090	5.34	< 0.0001
	3	0.027			0.038	0.065	6.02	< 0.0001
	4				0.027	0.027	3.16	0.003
Overall	1–3	0.165	4.02	0.0003				
	2–4				0.100			
	1–4					0.265		
Third-generation cephalosporin use								
Each month	4	0.116				0.116	2.75	0.009
	5	0.087			0.049	0.136	3.27	0.002
	6	0.058			0.057	0.115	3.70	0.0007
	7	0.029			0.048	0.077	3.91	0.0004
	8				0.032	0.032	2.75	0.009
Overall	4–7	0.290	2.75	0.009				
	5–8				0.186			
	4–8					0.476		
Fluoroquinolone use								
Each month	4	0.170				0.170	3.43	0.002
	5	0.085			0.071	0.156	3.37	0.002
	6				0.066	0.066	2.31	0.03
Overall	4–5	0.255	3.43	0.002				
	5–6				0.137			
	4–6					0.392		
Constant		–36.7	–4.42	0.0001				

^a^MRSA, methicillin-resistant *Staphylococcus aureus.* ^b^Past %MRSA as well as past use of these three antimicrobial drug classes had direct effects on %MRSA. These direct effects diminished the longer the lag time. ^c^Because every increase in %MRSA by the value 1 was followed the next month by a significant increase in %MRSA by the value 0.420, use of the three antimicrobial drug classes also had indirect effects on the %MRSA. As 0.420 is <1, these indirect effects necessarily vanished over time. As an example, decreasing indirect effects are only presented for a few months. There were substantial indirect effects of macrolide use up to month 8 (final coefficient for sum of both effects = 0.284), of third-generation cephalosporin use up to month 12 (final coefficient for sum of both effects = 0.499), and of fluoroquinolone use up to month 11 (final coefficient for sum of both effects = 0.440). ^d^Each month, the total effect of each class of antimicrobial on the %MRSA resulted from the sum of the direct and indirect effects. ^e^The estimated coefficients indicate the values by which the %MRSA would increase in response to an increase in 1 DDD per 1,000 patient-days for each of the three significant antimicrobial classes, when all other variables remain constant. Since the average figure for monthly patient-days at Aberdeen Royal Infirmary is 22,800, 10 DDD per 1,000 patient-days correspond to approximately 230 DDD per month or thirty 7- to 8-day antimicrobial courses. For example, an increase in macrolide use by 10 DDD per 1,000 patient-days on a certain month, or 30 more patients treated with a macrolide as compared with the previous month, would lead to a direct increase in %MRSA by 0.83, 1 month later, by 0.55, 2 months later and by 0.27, 3 months later. The total direct effect would therefore be evident after 3 months, amounting to an increase in %MRSA by the value 1.65. Additionally, %MRSA indirectly attributable to macrolide use would increase by the value 0.35 (i.e., 0.83 x 0.42) after 2 months and by 0.38 (i.e.. [0.83 x 0.42] + [0.55 x 0.42]) after 3 months. From the 4th month onwards, there would be no direct effect of macrolide use on the %MRSA, only ever-decreasing indirect effects that would practically disappear after 8 months (decreasing effects in months 5 to 8 not shown).

The sum of the direct and indirect effects of 10 DDD/1,000 patient-days or 30 more patients treated with a macrolide (Table 4) was an increase in %MRSA by the value 2.84 after 8 months. This change in antimicrobial drug use had more effect on the %MRSA in 1997 than in 2000. For example, in June 1997 the %MRSA was 3.6%. According to our model, an increase in macrolide use of 10 DDD/1,000 patient-days, or 30 more treated patients, made the %MRSA rise to 3.6 + 2.84 = 6.4% after 8 months or an 81% increase over June 1997. In June 2000, the %MRSA had reached 32.1%. An increase in macrolide use of 10 DDD/1,000 patient-days, or 30 more treated patients, made the %MRSA rise to 32.1 + 2.84 = 34.9% after 8 months or a 9% increase over June 2000. This observation suggests that antimicrobial drug use was a more important ecologic risk factor at the start of the outbreak than once MRSA had become endemic in the hospital. However, macrolide use kept increasing during the study period (Figure 2), which compensated for the decrease in the size of the effect of antimicrobial drug use on %MRSA. Similar effects were observed for third-generation cephalosporin and fluoroquinolone use, i.e., an increase of 10 DDD per 1,000 patient-days on a certain month or 30 more treated patients, resulted in an increase in %MRSA by 4.99 after 12 months for third-generation cephalosporins and by 4.40 after 11 months for fluoroquinolones.

The determination coefficient (R^2^) of the final model was 0.902, i.e., 90.2% of the variations of the monthly %MRSA from June 1997 to December 2000 were explained by the model. The model that did not take antimicrobial drug use into account (i.e., considered previous monthly %MRSA) had a lower determination coefficient (0.811) and over- or underestimated the monthly %MRSA by 7.93%. The model that took into account both previous monthly %MRSA and previous use of the three key classes of antimicrobial drugs, with a determination coefficient of 0.902, produced an average discrepancy of 2.84 percentage points with the observed %MRSA. Therefore, taking antimicrobial drug use into account helped to improve the precision in forecasting the monthly %MRSA by 64%, which is a clear indication that antimicrobial drug use has a substantial causal effect on the %MRSA.

We compared coresistance patterns of MRSA isolates from the outbreak (i.e., 1997–2000) and of MSSA from the same period (Table 1), which confirmed the consistency of the antimicrobial drug use included in the model. MRSA isolates from the outbreak period were almost always resistant to erythromycin, clindamycin, and ciprofloxacin, whereas MSSA isolates from the same period were resistant in 14.5%, 12.4%, and 35.5% of cases, respectively. Resistance of MRSA isolates to the other antimicrobial drugs tested never exceeded 11% and was lower than in MSSA isolates with the exception of mupirocin (6.1% in MRSA isolates, 1.9% in MSSA isolates).

**Figure 3:**
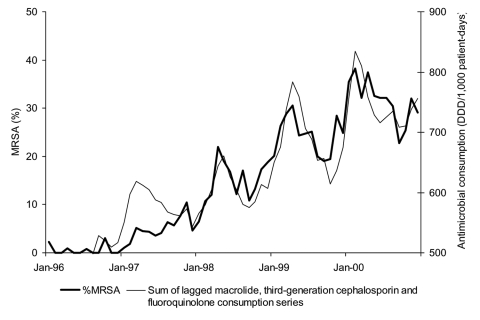
Evolution of the monthly % methicillin-resistant *Staphylococcus aureus* (MRSA) and monthly sum of lagged antimicrobial use as identified in polynomial distributed lag (PDL) model: macrolides (lags of 1 to 3 months), third-generation cephalosporins (lags of 4 to 7 months), and fluoroquinolones (lags of 4 and 5 months), Aberdeen Royal Infirmary, January 1996–December 2000.

Finally, a curve of the summed monthly use of macrolides, third-generation cephalosporins, and fluoroquinolones, which took into account their respective lags for direct effects, was constructed and plotted on the same graph as monthly %MRSA (Figure 3). This figure shows the striking parallel nature of the relationship between the lagged use of these specific antimicrobial classes and the %MRSA at Aberdeen Royal Infirmary, which confirms the findings visually.

## Discussion

For the first time, a powerful statistical model provides evidence of a strong temporal relationship between antimicrobial drug use and the varying prevalence of MRSA over time during an outbreak in a single hospital. The fact that only three classes of antimicrobial drugs, namely third-generation cephalosporins, fluoroquinolones, and macrolides, showed this relationship is not surprising. In the past, exposures to cephalosporins (26,27), fluoroquinolones (27–32), and macrolides (30) have been reported as patient risk factors for MRSA infection or colonization. And cephalosporin (4,8,33,34), fluoroquinolone (5,8,33), and macrolide use (8) have been reported as ecologic risk factors for high, or parallel variations of, MRSA prevalence or incidence. At Aberdeen Royal Infirmary, MRSA isolates were typically resistant to macrolides and fluoroquinolones (Table 1). Additionally, third-generation cephalosporins have poor activity against MRSA. At the same time, macrolides (clarithromycin and erythromycin), third-generation cephalosporins (mainly cefotaxime), and fluoroquinolones (essentially ciprofloxacin) were among the most used antimicrobial drugs at Aberdeen Royal Infirmary (Table 2), thus providing MRSA isolates with an ecologic advantage over other bacteria. Although the Aberdeen Royal Infirmary MRSA isolates were almost always resistant to clindamycin, use of lincosamides was among the lowest, which might explain why it did not appear as a risk factor in the multivariate model.

In addition to antimicrobial drug use, the final model also included the %MRSA observed 1 month before. As mentioned, we did not uniformly look for MRSA colonization. The pressure attributable to MRSA-colonized patients is a known risk factor for MRSA acquisition (8,35), which in turn affects the number of MRSA infections and the %MRSA in *S. aureus* from clinical samples. We therefore think that the %MRSA observed 1 month before is a surrogate for the pressure attributable to MRSA-positive patients during the past month.

The study was an ecologic and uncontrolled observational study in a single hospital. Selection bias was unlikely because data represented all hospitalized patients. Information bias was unlikely because data were not specifically collected for our study but for other purposes, i.e., routine clinical microbiologic diagnosis for *S. aureus* data and pharmacy accounting for antimicrobial drug use data. Confounding factors cannot be excluded but are unlikely for two reasons. First, as a result of the applied modeling strategy, the monthly variation in %MRSA not explained by the model (9.8%) was random. Therefore, the role of any possible unidentified confounding variable is thought to be minimal. Second, infection control policies, including measures such as barrier nursing, single room isolation, and eradication of carriage have consistently been applied to all MRSA patients during the study period, although a shortage of single rooms often necessitated several MRSA-positive patients being assigned to a single nurse. Staff MRSA carriers were not actively sought, but use of gloves and hand washing, as appropriate, were constantly emphasized. Active patient contact tracing was applied, when possible, but environmental cleaning relied on standard cleaning schedules rather than environmental screening and targeted interventions. This policy was in line with national guidelines (36). The relationships between antimicrobial drug use and the %MRSA were unlikely to be attributable to chance because p values in the model were low. Additionally, the cause-effect relationships in the model were validated by their temporal nature (i.e., use of macrolides, third-generation cephalosporins, and fluoroquinolones always preceded %MRSA). Additionally, for each of these antimicrobial drug classes, the effect of antimicrobial drug use on the %MRSA was directional (i.e., an increase in use resulted in increased %MRSA and a decrease in use resulted in decreased %MRSA). In contrast, variations in glycopeptide use followed variations in %MRSA with an average delay of 1 month (coefficient = 0.45).

The relative importance of antimicrobial drug use compared to cross-transmission or changes in the patient case-mix could not be assessed. In ecologic analyses with aggregated data, additional data, such as volumes of medicated soaps or alcoholic solutions used for hand hygiene, could be used as surrogates for infection control practices; however, these data were not available. As in many hospitals, patient-level data were not available, which is why we modeled aggregated microbiology and pharmacy data. Models that use patient-level data on both antimicrobial drug exposure and MRSA may reach different conclusions. For example, the risk period for a patient for acquiring MRSA then developing an infection would be limited to hospital stay, which is generally short and rarely longer than 1 month. However, our model showed that a delay of several months was sometimes necessary to observe an ecologic effect of antimicrobial drug use on the %MRSA. This result is difficult to interpret since it means that antimicrobial drug exposure of some patients on a certain month has an impact on MRSA infections in other patients several months later. Since antimicrobial drug use data are based on dispensations to the wards, antimicrobial drugs can be stocked in the wards and used over several months. However, pharmacy data showed that antimicrobial agents were dispensed several times per ward each month, making this explanation unlikely. Another explanation could be that the increase in antimicrobial drug use would contribute to increasing the size of the reservoir of MRSA carriers. First, MRSA clones would be selected in antimicrobial drug-exposed patients. Then, the size of the reservoir of MRSA carriers would gradually increase through the spread of these MRSA clones to other patients, hospital staff, and the environment. This increase would become evident in clinical samples after several months when the MRSA reservoir reached a certain size. For fluoroquinolones, this hypothesis is supported by the results of Bisognano et al. (37) and Harbarth et al. (31). These authors showed that sub-MIC levels of ciprofloxacin increase adhesion of quinolone-resistant MRSA, which could explain persistent MRSA carriage and failure of mupirocin treatment in patients who received a fluoroquinolone. Antimicrobial drug use and cross-transmission probably work together to influence the %MRSA, and if all cross-transmission were to stop after implementing a very successful control program, the relationship between fluoroquinolone use and %MRSA would most probably disappear. Further research is needed to confirm this hypothesis and, more generally, to understand why long delays are also observed for other antimicrobial drugs, e.g., third-generation cephalosporins.

At Aberdeen Royal Infirmary, antimicrobial drug prescribing is overseen by an antibiotic committee, which provides and regularly updates a joint hospital-community antibiotic policy and stewardship program (38). Antimicrobial prescribing audits are performed periodically, but changing prescribing practices to control MRSA has not been attempted. Third-generation cephalosporin prescribing was addressed previously during an outbreak of *Klebsiella pneumoniae* displaying extended-spectrum β-lactamase activity (39). With the implementation of the British Thoracic Society guidelines for treatment of community-acquired pneumonia (40), macrolide and third-generation cephalosporin (mainly cefotaxime) prescribing has increased, which has been paralleled by the increase in MRSA. As the Aberdeen MRSA clones are relatively susceptible, a policy of therapeutic substitution has been implemented in MRSA problem areas, starting with the replacement of cephalosporins by non–β-lactam antimicrobial drugs in surgical prophylaxis. The increase in fluoroquinolone prescribing has not been explained, but audits indicate that it is commonly used to treat serious nosocomial gram-negative infection (38).

Our study showed a quantifiable, temporal relationship between use of three classes of antimicrobial drugs (macrolides, third-generation cephalosporins, and fluoroquinolones) and the %MRSA. Because the study was performed in one hospital during an outbreak in which two predominant strains were circulating, it might not apply to other hospitals. Nevertheless, the use of antimicrobial drugs other than anti-staphylococcal penicillins and to which the MRSA outbreak strains are resistant might be a factor that would promote the outbreak. Moreover, the ecologic effect of antimicrobial drug use was confirmed (i.e., current antimicrobial drug use might have an effect on resistance in future patients). The effect of antimicrobial use on the %MRSA was greatest when the outbreak started and decreased when the %MRSA increased. Large decreases in antimicrobial drug use would have been needed to affect MRSA once it had become endemic. However, programs to control prescriptions of selected antimicrobial drug classes could represent an adjunct measure to active surveillance cultures and barrier precautions for the control of clonal outbreaks of MRSA, which has proved difficult and expensive.

## Appendix

### Polynomial Distributed Lag (PDL) model

A PDL model was built to detect and quantify the lagged effects of antimicrobial use on the % methicillin-resistant *Staphylococcus aureus* (MRSA). In a PDL model, the relationship between the dependent variable (resistance) and the independent variables (past resistance and antimicrobial use) should evolve smoothly over time, through the use of "polynomial lags." The optimum PDL model was arrived at by the "general-to-specific" econometric methodologic characteristics. This meant that, initially, many possible independent variables were included in the model, some of which were ultimately found to be irrelevant. Additionally, for all the independent variables, lags of up to 8 months were initially included to identify direct effects. The initial dynamic regression model with PDLs considering %MRSA series as the dependent variable and several antimicrobial drug use series as explanatory series was the following:


_
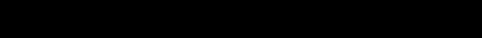
_


with PDL restrictions on the coefficients of antimicrobial use and where MAC means macrolide use, 3GC third-generation cephalosporin use, FQ fluoroquinolone use and PIB use of penicillins with β-lactamase inhibitors. The model was initially estimated on the full study period, i.e., January 1996–December 2000, using a degree *q_j_* of the polynomial equal to 3. The estimated model was compatible with normal white noise errors (absence of autocorrelation and absence of heteroskedasticity), and no signs of nonmodeled nonlinearities were seen.

This initial model was then simplified to eliminate irrelevant antimicrobial drug uses and unnecessary lags. In the first steps of the simplification, all antimicrobial drugs were kept in the model, and the simplification took the form of reducing the order of the polynomial and eliminating unnecessary lags. Along this process, use of penicillins with β-lactamase inhibitors did not appear to play a significant role and was eliminated from the model. We also tried to introduce use of each of the other antimicrobial drug classes that showed a relationship in Table 3; however, none appeared to play an important role, and they were not included in the model. Further simplification of the distributed lags of macrolide use, third-generation cephalosporin use, and fluoroquinolone use of the %MRSA itself led to a model in which, through CUSUM and CUSUMSQ statistics, a structural change was detected around the middle of 1997. Application of the Chow test located the change in June 1997. The %MRSA was virtually zero in 1996 and started to increase at the beginning of 1997, which was consistent with the fact that the MRSA epidemic strain, resistant to macrolides and fluoroquinolones, only became predominant in 1997. In 1996, 56% and 50% of MRSA isolates were resistant to erythromycin and ciprofloxacin, respectively, whereas these percentages suddenly rose to 92% and 89%, respectively, in 1997. Data before June 1997 were considered as not being part of the outbreak and were therefore not included in the final model. The validity of the simplified, final model from June 1997 onwards was checked by a battery of specification and diagnostic tests to verify the absence of autocorrelation of residuals, absence of heteroskedasticity, normality of residuals, absence of nonmodeled nonlinearities and absence of structural change.

The basic measure of forecasting quality, Root Mean Squared Error of Forecast (RMSEF) was also computed, which provided an average measurement of the amount by which the model over- or underestimated the %MRSA. RMSEF was calculated for a model without antimicrobial drug use (based on past %MRSA only) and compared with that of the final model, which included antimicrobial drug use.
